# Coupling Electro-Fenton and Electrocoagulation of Aluminum–Air Batteries for Enhanced Tetracycline Degradation: Improving Hydrogen Peroxide and Power Generation

**DOI:** 10.3390/molecules29163781

**Published:** 2024-08-09

**Authors:** Zhenghan Zhou, Wei Wei, Houfan Wu, Haoyang Gong, Kai Zhou, Qiyuan Zheng, Shaogen Liu, Ling Gui, Zhongqi Jiang, Shuguang Zhu

**Affiliations:** 1 School of Environment and Energy Engineering, Anhui Jianzhu University, Hefei 230601, China; sahara@stu.ahjzu.edu.cn (Z.Z.); whf991009@126.com (H.W.); liushgen@mail.ustc.edu.cn (S.L.); 15656990852@163.com (L.G.); zqjiang0411@163.com (Z.J.); 2 Anhui Provincial Key Laboratory of Environmental Pollution Control and Resource Reuse, Hefei 230061, China; 3 Key Laboratory of Water Pollution Control and Wastewater Reuse of Anhui Province, Hefei 230061, China; 4 An Hui Shun Yu Water Co., Ltd., Hefei 230601, China; chenpenggzun@163.com (K.Z.); zhangq19901018@163.com (Q.Z.); 5 Hefei Water Supply Group Co., Ltd., No. 70, Tunxi Road, Hefei 230011, China; gonghaoyang1998@126.com; 6 Energy Saving Research Institute, Anhui Jianzhu University, Hefei 230601, China; 7 Engineering Research Center of Building Energy Efficiency Control and Evaluation, Ministry of Education, Anhui Jianzhu University, Hefei 230601, China

**Keywords:** aluminum–air batteries, electro-Fenton, electrocoagulation, tetracycline removal

## Abstract

Electro-Fenton (EF) technology has shown great potential in environmental remediation. However, developing efficient heterogeneous EF catalysts and understanding the relevant reaction mechanisms for pollutant degradation remain challenging. We propose a new system that combines aluminum–air battery electrocoagulation (EC) with EF. The system utilizes dual electron reduction of O_2_ to generate H_2_O_2_ in situ on the air cathodes of aluminum–air batteries and the formation of primary cells to produce electricity. Tetracycline (TC) is degraded by ·OH produced by the Fenton reaction. Under optimal conditions, the system exhibits excellent TC degradation efficiency and higher H_2_O_2_ production. The TC removal rate by the reaction system using a graphite cathode reached nearly 100% within 4 h, whereas the H_2_O_2_ yield reached 127.07 mg/L within 24 h. The experimental results show that the novel EF and EC composite system of aluminum–air batteries, through the electroflocculation mechanism and ·OH and EF reactions, with EC as the main factor, generates multiple •OH radicals that interact to efficiently remove TC. This work provides novel and important insights into EF technology, as well as new strategies for TC removal.

## 1. Introduction

In recent years, there has been a growing concern over environmental issues stemming from pharmaceuticals and personal care products (PPCPs) [1,2]. Tetracycline (TC), a commonly found PPCP, finds extensive use in diverse sectors, including human medicine, animal husbandry, and aquaculture [3,4]. However, due to its limited absorption by humans and animals, significant amounts of residual TC are inevitably released into the natural environment. Its accumulation in ecosystems fosters the emergence of drug-resistant bacteria, posing a grave threat to human health [5,6,7,8]. Various methods have emerged to address TC contamination [9], including adsorption techniques [10], membrane separation [11], photocatalytic oxidation [12,13,14], ozonation [15], electrocoagulation (EC) [16], and electro-Fenton (EF) processes [17]. Electrochemical technologies, encompassing EC and Fenton methods, have demonstrated notable efficacy in removing TC. 

Aluminum–air batteries are a novel form of energy batteries, employing an aluminum anode, air cathode, and electrolyte. Using an aluminum plate as the electrode presents several advantages, including the abundant reserves, affordability, and non-toxic nature of aluminum. Furthermore, the aluminum salt generated within the cell is an efficient coagulant with potential applications in water pollutant removal [17,18]. EC, recognized as a highly effective water treatment technology, is widely applied for eliminating various waterborne contaminants, including antibiotics [19,20,21,22,23,24]. However, the EC process necessitates an external power source, resulting in significant power consumption and increased operational costs. In contrast, aluminum–air battery EC technology has emerged, integrating the principles of aluminum–air batteries and EC. This innovative approach leverages the strengths of both techniques, notably producing flocculants without requiring an external power supply. Previous studies have demonstrated [25,26] its efficacy in addressing a broad spectrum of water pollutants.

However, although EC alone eliminates TC primarily through the physical mechanism of flocculation, it lacks a degradative effect and generates residual sludge that poses environmental hazards. In contrast, the Fenton process, regarded as an advanced oxidation method, mineralizes complex pollutants into smaller benign molecules generated through the action of hydroxyl radicals (·OH) formed through the Fenton reaction (Equation (1)) [27,28,29,30,31,32]. Moreover, the continual regeneration of Fe^2+^ from reduced Fe^3+^ ensures a self-sustaining catalytic cycle (Equation (2)) [33]. Although introducing external H_2_O_2_ is common in conventional Fenton processes, this inherently restricts the technique’s broader application. Furthermore, potential challenges exist in the storage and transportation of Fenton reagents [34].

H_2_O_2_ + Fe^2+^ → Fe^3+^ + ·OH + OH^−^ (1)

Fe^3+^ + H_2_O_2_ → HO_2_· + Fe^2+^ + H^+^ (2)

EF technology enables in situ H_2_O_2_ generation by reducing oxygen (O_2_), eliminating the need for external addition. However, this process necessitates an additional power supply, inevitably leading to increased operational expenses. In the context of the reaction within aluminum–air batteries, O_2_ undergoes dual reduction pathways on the air cathodes: the two-electron and four-electron pathways (Equations (3) and (4)) [35,36].

O_2_ + 2e^−^ + 2H^+^ → H_2_O_2_ (3)

O_2_ + 4e^−^ + 4H^+^ → 2H_2_O (4)

Theoretically, the cell can autonomously produce H_2_O_2_ via the two-electron reduction pathway of O_2_ without requiring an external power source. Thus, the Fenton reaction can be initiated by incorporating an iron-based catalyst within the reactor, producing ·OH radicals. These radicals play a crucial role in degrading diverse pollutants, thereby significantly reducing energy consumption. The primary O_2_ reduction pathway depends on the composition of the catalytic layer within the air cathode [37,38,39]. Carbon-based materials, including carbon cloth [40], graphite rods [41], and modified graphite [42], are frequently selected for H_2_O_2_ synthesis due to their excellent conductivity, robust stability, and cost-effectiveness.

This paper explores the utilization of aluminum–air batteries for the in situ production of flocculants and H_2_O_2_ to generate ·OH through the Fenton reaction. This approach facilitates the synergistic action of EF and EC in eliminating TC without requiring an external power supply. Three carbon-based materials—activated carbon (AC), carbon black (CB), and graphite (G)—serve as catalysts in preparing the air cathodes. The performance of these different cathodes is comprehensively analyzed, comparing the H_2_O_2_ synthesis and power generation of aluminum–air batteries using alternative air cathodes. Furthermore, this investigation determines the TC removal rate under various conditions to ascertain the optimum removal parameters.

## 2. Results and Discussion

### 2.1. Performance of Different Air Cathodes

#### 2.1.1. Morphology and Porosity

**Figure 1:**
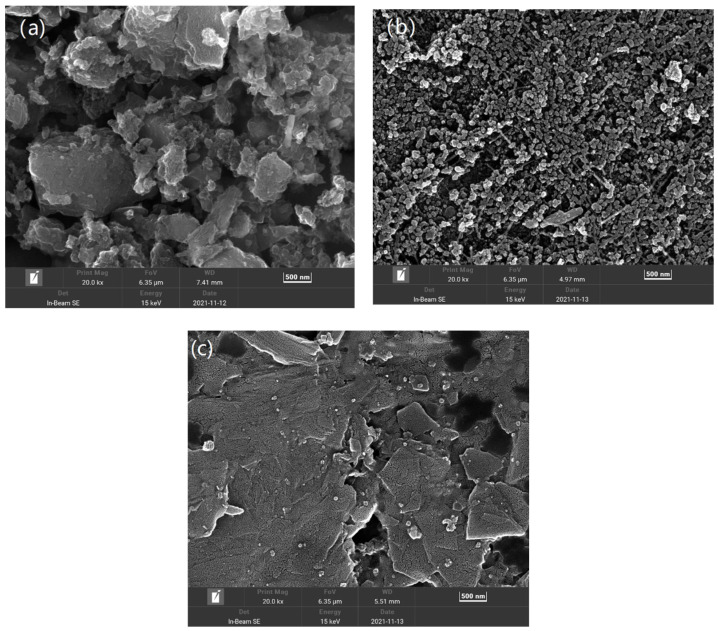
SEM images of (**a**) AC, (**b**) CB, and (**c**) G cathodes at a magnification of 15 K.

Figure 1 illustrates the surface morphology of the CL of the three air cathodes. The AC cathode exhibits large irregular aggregates and some internal irregular pores. In contrast, the CB cathode shows closely packed point-like particles. The graphite cathode comprises whole flake graphite with a smooth surface and relatively few visible pores.

**Figure 2:**
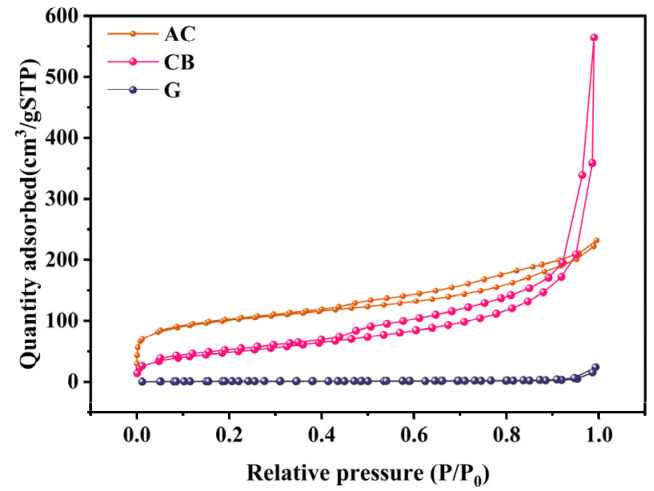
N_2_ adsorption/desorption isotherms of different carbon–PTFE powders.

Figure 2 illustrates the N_2_ adsorption isotherms of the carbon–PTFE powders. Based on the IUPAC classification, the adsorption isotherms of AC-PTFE powder fell into type IV and displayed noticeable hysteresis at higher P/P_0_ values. This hysteresis phenomenon arises from capillary condensation within the mesoporous structure [43]. Specifically, the hysteresis loop observed on the AC adsorption isotherm was classified as type H4, indicating the presence of slit-like mesoporous systems [44]. CB-PTFE powder’s adsorption/desorption isotherms corresponded to type V in the IUPAC classification, indicating that the CB cathode engages in mesoporous adsorption with a subdued affinity. Furthermore, type-H3 hysteresis rings exhibit sharper shapes at elevated relative pressures, suggesting a higher abundance of mesopores and possibly even macropore adsorption [45]. The isotherm of the G cathode aligned with type III, indicating that graphite operates as a microporous adsorbent with limited affinity [46].

Table 1 summarizes the pore data of the three carbon–PTFE powders. The data indicate that AC-PTFE powder exhibited the highest BET surface area (355.3996 m^2^/g), followed by CB (177.8102 m^2^/g), with the graphite cathode having the lowest specific surface area (2.9440 m^2^/g). The micropore volume of the AC cathodes (0.060737 cm^3^/g) was markedly higher than that of the CB (0.000224 cm^3^/g) and G (0.000041 cm^3^/g) cathodes. Moreover, compared with the CB and G cathodes, the average aperture size of the AC cathode was smaller. This indicated abundant pores and more microporous structures in the AC cathode compared with the CB and G cathodes. The specific surface area and pore volume of G-PTFE powder were much smaller than those of the other two powders.

#### 2.1.2. Electrochemical Analysis

**Figure 3:**
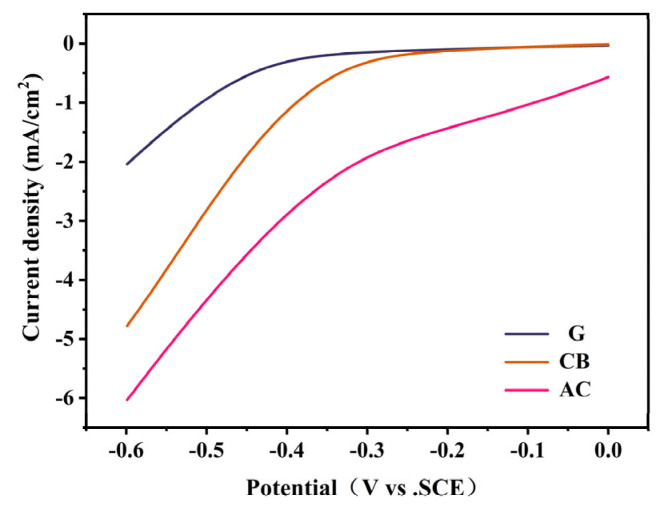
Linear sweep voltammetry of three air electrodes.

Catalytic activity for O_2_ reduction (ORR) was assessed through linear sweep voltammetry (LSV) tests spanning 0 V to 0.6 V (Figure 3). The current response of the AC cathode was notably superior to that of the CB and G cathodes, underscoring the AC cathode’s heightened ORR catalytic efficacy compared to the other two cathodes. As the voltage decreased to −0.25 V, CB and G exhibited similar current curves, yet the current of the CB cathode exhibited a pronounced escalation relative to the G cathode as the voltage became more negative. Within the 0 V to −0.25 V range, the CB and G cathodes displayed markedly diminished catalytic activity. In summary, the ORR catalytic performance of the three cathodes ranks in the order AC > CB > G.

**Figure 4:**
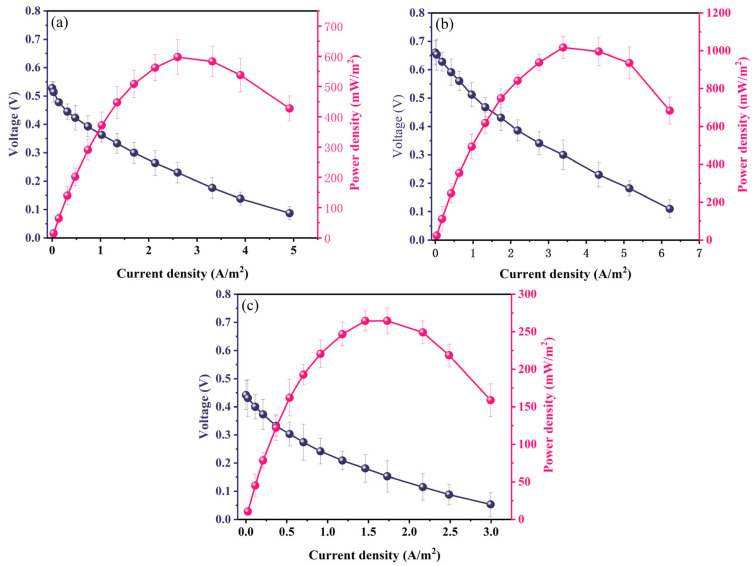
Power density and polarization curves of aluminum–air batteries using different cathodes: (**a**) AC, (**b**) CB, and (**c**) G cathodes.

Similar trends were evident in the polarization and power density curves (Figure 4). The polarization curves illustrated that the open circuit voltages (OCVs) for cells employing AC, CB, and G cathodes were measured at 0.660 V, 0.28 V, and 0.422 V, respectively. Notably, the G cathode exhibited a faster rate of voltage decay. The power density curve underscored these differences, with the AC cathode achieving a maximum power density of 1016.95 mW/m^2^, followed by the CB cathode with 597.74 mW/m^2^ and the G cathode with the lowest maximum power density of 264.51 mW/m^2^. These variations in performance can be attributed to dissimilarities in the surface morphology and pore structure. During the cathodic reaction process, O_2_ initially diffuses to the surface with macropores or mesopores, penetrating the internal micropores for the reaction [45]. The AC cathode, owing to its larger specific surface area and more abundant distribution of micropores, provides more reaction sites for ORR with O_2_ [47]. This advantageous configuration contributes to the AC cathode’s superior electricity generation performance, significantly enhancing its power output. In contrast, the CB and G cathodes, characterized by smaller specific surface areas, exhibit diminished power densities.

#### 2.1.3. Performance of H_2_O_2_ Production

**Figure 5:**
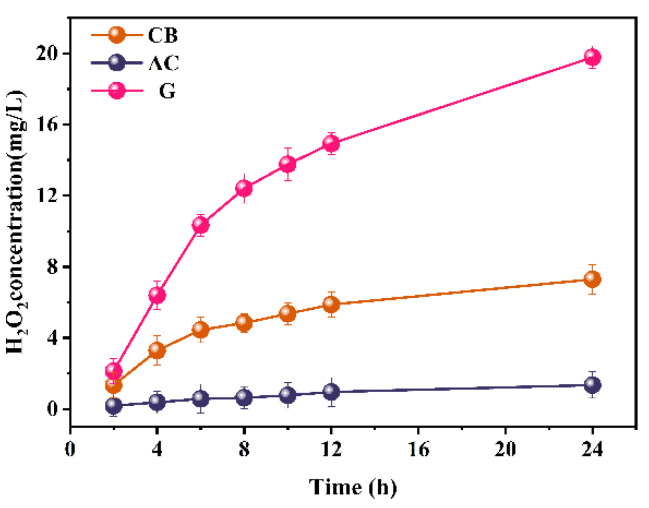
H_2_O_2_ concentration of aluminum–air batteries using AC, CB, and G cathodes.

The H_2_O_2_ concentration was measured in the cells after 2, 4, 6, 8, 10, 12, and 24 h (Figure 5). Notably, the cell with a G cathode exhibited the most effective H_2_O_2_ production, closely followed by the cell with a CB cathode, whereas the cell with the AC cathode yielded the lowest levels. After a 24 h reaction, the H_2_O_2_ concentrations in the AC, CB, and G cathodes were measured at 1.35, 7.30, and 19.78 mg/L, respectively. This trend contradicted the observed power output and catalytic activity trends. This discrepancy suggests that O_2_ favors four-electron reduction within the AC air cathode and two-electron reduction within the G cathode. This behavior can be attributed to the larger surface area and higher concentration of micropores within the AC cathode. Consequently, the cell employing the AC cathode demonstrated higher electron utilization efficiency and superior electricity generation performance, albeit with lower H_2_O_2_ production. Conversely, the G cathode exhibited elevated H_2_O_2_ output but poorer power generation. The difference in H_2_O_2_ yield from the G cathode compared to the others is related to the structural characteristics of the three catalyst materials. According to previous studies, during the reduction of O_2_, the oxygen molecules first diffuse to the surfaces of macropores and mesopores before moving into the micropores for the ORR reaction. Our analysis indicates that the AC cathode has a rich microporous structure, providing more ORR sites, which enhances the efficiency of oxygen reduction. As a result, oxygen tends to undergo a four-electron transfer reduction to H_2_O, leading to better power generation performance but lower H_2_O_2_ yield for the AC cathode. In contrast, the graphite cathode has a sheet-like surface structure with fewer pores and a larger average pore size, primarily composed of macropores and mesopores. This structure favors a two-electron pathway for the ORR, resulting in higher H_2_O_2_ production but slightly lower power generation performance for the graphite cathode. The CB cathode has an average pore size between the two, with a predominance of mesopores, allowing it to support both the two-electron and four-electron ORR pathways. 

Furthermore, the data indicate a rapid rate of H_2_O_2_ production within the first six hours, which decelerates over time. This trend is particularly pronounced in the G cathode and may be attributed to the gradual accumulation of H_2_O_2_ within the electrolyte, leading to a decrease in the rate of O_2_ reduction.

Overall, the cell employing the AC cathode excelled in its power generation performance, whereas the cell with the G cathode demonstrated optimal H_2_O_2_ production. However, the degradation of TC necessitates substantial hydroxyl radicals. Therefore, graphite was employed as the air cathode catalyst in subsequent experiments.

### 2.2. Removal of TC

**Figure 6:**
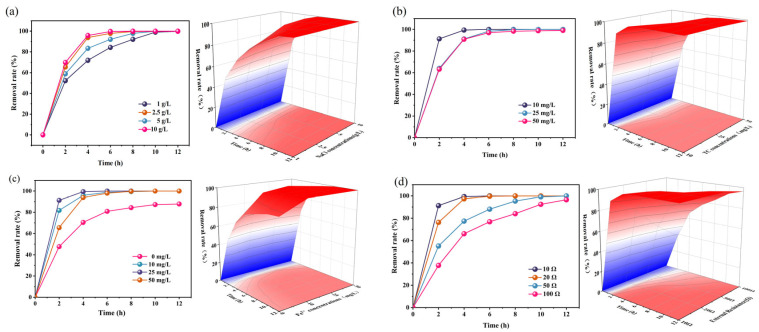
TC removal rates for different NaCl concentrations (**a**), initial TC concentrations (**b**), Fe^2+^ concentrations (**c**), and external resistance (**d**).

Figure 6 depicts the TC removal rate using aluminum–air batteries with a G cathode under varying parameters, including the electrolyte concentration, initial TC concentration, Fe^2+^ concentration, and external resistance. The TC removal rate consistently reached nearly 100% across all NaCl concentrations (Figure 6a). However, notably enhanced TC removal efficiency was observed with increasing NaCl concentration. This phenomenon can be attributed to higher NaCl solution concentrations elevating the electrolyte conductivity [48]. Consequently, the electron transfer rate within the system increases, boosting the production rate of both flocculants and H_2_O_2_. Furthermore, Cl^−^ ions within the electrolyte disrupt the corrosion protection layer on the aluminum sheet’s surface, effectively lowering the cell’s ohmic resistance [49]. Moreover, the initial TC concentration exerted a minor influence on TC removal efficiency (Figure 6b), as TC is ultimately removed from the solution regardless of its initial concentration.

An analysis of the TC removal efficiency at varying concentrations of Fe^2+^ revealed that the influence of the Fe^2+^ concentration on TC removal rates is intricate (Figure 6c). Without Fe^2+^ (control group), the TC removal rate reached 87.8% after 12 h. However, introducing Fe^2+^ yielded a notable enhancement in TC removal efficiency, surpassing 99% within 6 h. 

The relationship between the Fe^2+^ concentration and TC removal rate is nuanced. When the Fe^2+^ concentration is too low, the Fenton reaction’s effectiveness diminishes. Conversely, excessively high Fe^2+^ concentrations can lead to the scavenging of ·OH radicals within the solution. This scavenging effect competes with TC (as described in Equation (5)), consequently lowering the TC removal rate [50]. The balance lies between these extremes; hence, both excessively high and low Fe^2+^ concentrations are unfavorable for TC removal. Nonetheless, effective TC removal was observed across different Fe^2+^ concentrations.

Fe^2+^ + ·OH → Fe^3+^ + 3OH^−^ (5)

External resistance significantly impacts TC removal. A pronounced increase in the TC removal rate was observed with diminishing external resistance (Figure 6d). As the external resistance was decreased from 100 Ω to 10 Ω, the TC removal rate surged from 37.76% to 91.27% after 2 h. Notably, at an external resistance of 10 Ω, the TC removal rate exhibited the swiftest progression, reaching 99.37% within 4 h. In contrast, when the external resistance was set at 100 Ω, the TC removal rate was only 96.48% after 12 h. This phenomenon is closely tied to the circuit’s current density. 

**Figure 7:**
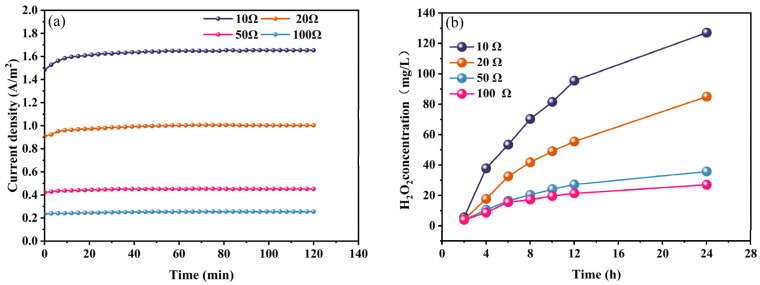
(**a**) Current density; (**b**) H_2_O_2_ production versus time of Al–air batteries at different external resistance levels.

The cell’s current density increased from 0.256 A/m^2^ to 1.653 A/m^2^ as the external resistance decreased from 100 Ω to 10 Ω (Figure 7a). Correspondingly, the H_2_O_2_ concentration increased from 26.96 mg/L to 127.07 mg/L after 24 h. Following Faraday’s Law, a heightened current density mitigates the competition between EC and EF reactions, leading to more comprehensive responses. This, in turn, results in enhanced pollutant removal efficacy [51].

### 2.3. Mechanism of TC Removal

**Figure 8:**
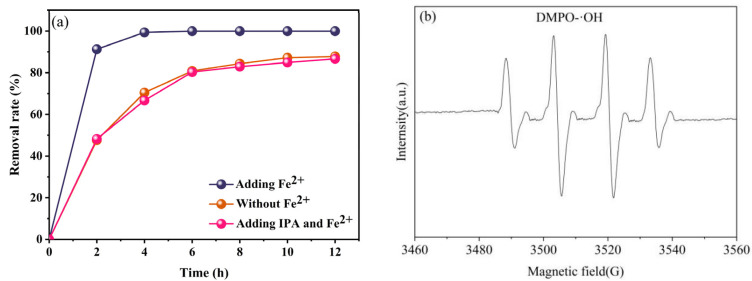
(**a**) Removal rate of TC in the different systems. (**b**) EPR spectrum of DMPO-⋅OH.

We conducted targeted experiments to investigate the mechanism underlying TC removal in the aluminum–air battery system by capturing hydroxyl radicals. For this purpose, isopropanol (IPA), known for its rapid reaction rate with ·OH, was employed as a free radical scavenger [52]. We established three distinct experimental groups: one with the addition of 25 mg/L Fe^2+^ as a catalyst, another with the joint presence of Fe^2+^ and IPA, and the third without the inclusion of either Fe^2+^ or IPA. The introduction of Fe^2+^ led to TC removal rates surpassing 99% after a 4 h reaction. However, upon the addition of IPA (Figure 8a), the TC removal rate decreased to 86.64%. This outcome highlights IPA’s capability to inhibit the activity of ·OH within the system. Notably, this effect mirrors the TC removal rate trend observed in the absence of Fe^2+^, wherein TC removal plateaued at 87.80%. To verify the free radical trapping results, the electron spin resonance (EPR) technique was applied to monitor the signal of ·OH, with 5, 5-dimethyl-1-pyrroline-N-oxide (DMPO) as the probe. A significant DMPO-·OH signal was observed in the aluminum–air battery system (Figure 8b), confirming that ·OH played a vital role in TC reduction. The results indicate that the removal rate of TC in the single aluminum–air fuel EC system was significantly lower than that in the EF and EC coupling system of aluminum–air batteries. This is due to EC only removing TC by physical action, while free radicals in the coupled system degrade TC into small molecules.

**Figure 9:**
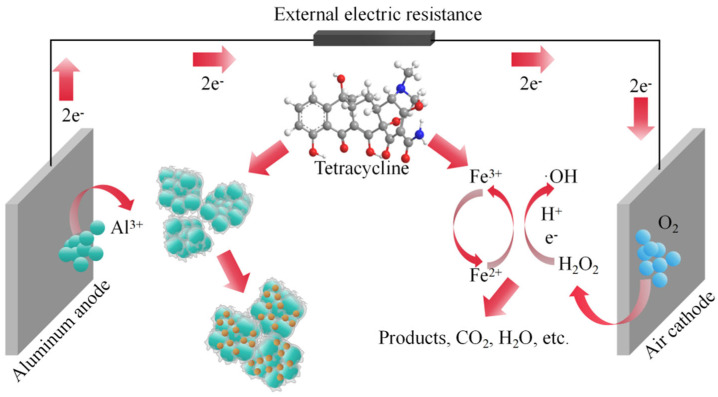
Mechanism of TC pollutant degradation by the EF and EC composite system.

Based on the collective study findings, the reaction mechanism for the removal of TC is proposed in Figure 9. EF and EC form a combined electrochemical system, supplemented by electroflocculation and accompanied by electron transfer, adsorption, coprecipitation, and other processes. The novel EF and electroflocculation composite system of aluminum–air batteries relies on metal valence state conversion and electron transfer to produce efficient synergies through electroflocculation, ·OH reactions, and EF reactions, a reaction mechanism with electroflocculation as the main factor and multiple •OH radicals interacting with each other to efficiently remove TC. 

**Figure 10:**
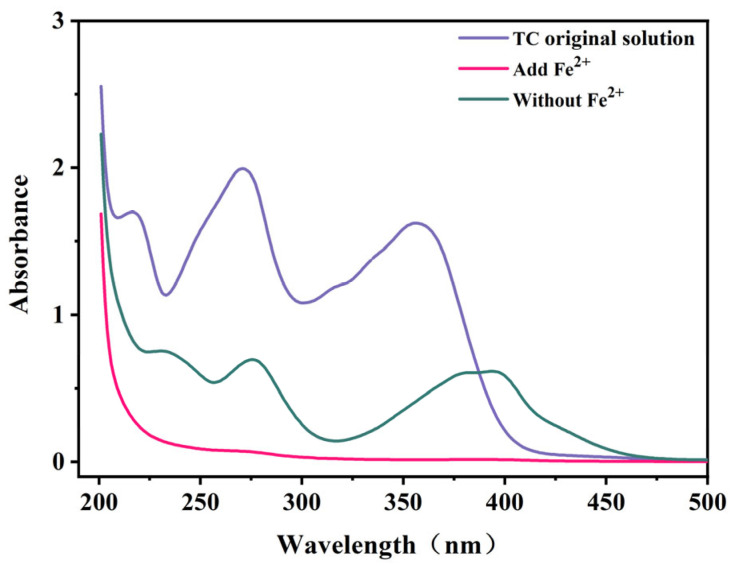
UV–vis spectra during the degradation of TC in different systems.

To further elucidate the mechanism underlying TC degradation and removal, UV–vis spectra were used to measure the TC absorbance in various systems. The original TC solution exhibited a primary absorption peak around 357 nm (Figure 10), indicative of the presence of a benzene ring in the TC structure [53]. Additionally, a shoulder peak emerged near 270 nm, denoting the existence of a naphthalene ring [54].

Upon the addition of Fe^2+^, the absorption peaks vanished, providing conclusive evidence of complete TC degradation. However, when Fe^2+^ was absent, two prominent absorption peaks persisted after 12 h. This lends support to the effectiveness of the aluminum–air batteries in TC degradation through the action of ·OH. Based on the fundamental principles of advanced oxidation, ·OH can mineralize TC into smaller molecules, leading to the formation of CO_2_ and water [55,56]. Conversely, in the absence of added Fe^2+^, TC primarily undergoes removal through EC without concurrent degradation.

## 3. Materials and Methods

### 3.1. Air Cathode Fabrication

Air cathodes are crucial components of aluminum–air batteries, comprising a catalytic layer (CL), a gas diffusion layer (GDL), and a current collector. To prepare the GDL, CB powder was dispersed in ethanol using an ultrasonic bath for 10 min. PTFE emulsion was slowly added as a binder, with a CB-to-PTFE mass ratio of 3:7. After 30 min of ultrasonic stirring, the mixture was heated to 80 °C in a water bath until it formed a paste. The paste was applied to one side of a stainless-steel mesh, resulting in a 0.45 mm gas diffusion layer.

For the CL, a paste was prepared using carbon powder (CB, AC, G) and PTFE emulsion with a mass ratio of 3:1, following the same method as described above. The resulting paste was rolled onto the other side of the stainless-steel mesh to form a 0.9 mm air cathode. Finally, the air cathode was calcined at 340 °C for 25 min. Three air cathodes were prepared and labeled AC, CB, and G. 

### 3.2. Setup and Operation

The experimental setup comprised an 18 cm × 3 cm × 18 cm rectangular reactor that was hollow and cylindrical, with a height of 3 cm and a diameter of 15 mm. The front and back of the container were constructed using square plexiglass panels, whereas the anode was crafted from aluminum. The electrolyte was a 5 g/L sodium chloride (NaCl) solution. The front and back sides were composed of square plexiglass panels, and the anode comprised aluminum plates. The H_2_O_2_ production and power generation performance were assessed using various air cathodes within a static battery system. FeSO_4_ catalyzed the generation of ·OH for TC degradation. The solution movement was regulated by a peristaltic pump. The pH of the electrolyte was adjusted using hydrochloric acid and a sodium hydroxide solution. Samples were collected every 2 h from the upper hole and underwent filtration using a 0.45 µm filter membrane. 

### 3.3. Material Characterization

The morphology of the CL surface was observed using a scanning electron microscope (SEM). The specific surface characteristics and porosity of the carbon–PTFE powder were automatically assessed using a specialized analyzer that measured nitrogen adsorption and desorption (BET analysis). The specific surface area of the powder was also determined using this method. Additionally, pore parameters of the sample were analyzed using the BJH model. 

### 3.4. Electrochemical and Chemical Analyses

The voltage of the battery was collected using a data acquisition system. The maximum power density was evaluated by measuring polarization power density curves, which involved changing external resistances between 1000 Ω and 1 Ω. Linear sweep voltammetry (LSV) measurements were conducted using an electrochemical workstation, with the air cathode serving as the working electrode; a saturated calomel electrode (SCE), which is a reference electrode using a saturated potassium chloride solution, as the reference electrode; and a platinum sheet as the counter electrode. The potential scanning ranged from 0 V to −0.6 V at a scanning speed of 0.001 V/s.

To determine the H_2_O_2_ content, a TU-1950 double-beam UV–Vis spectrophotometer from Beijing Purkinje General Instrument Co., Ltd. (Beijing, China), was used at 385 nm with potassium titanium oxalate as the chromogenic agent. Similarly, the TC concentration was measured at 357 nm using a spectrophotometer. All experiments were duplicated, and the mean is reported. 

## 4. Conclusions

Air cathodes were prepared using three carbon-based catalysts: AC, CB, and G. The impacts of these cathodes on H_2_O_2_ production and power generation were assessed, with the G cathode yielding the highest amount of H_2_O_2_, whereas the AC cathode exhibited superior power generation due to its larger surface area and more numerous ORR sites. This study also explored TC removal through aluminum–air batteries with G catalysts under different conditions. After four hours, the removal rate of TC reached nearly 100% and was achieved through the synergistic effect of EC and EF. Using free radical capture experiments, we further confirmed that ·OH played a significant role and degraded TC molecules through mineralization.

## Figures and Tables

**Table 1:** Porous structural characteristics of carbon–PTFE powders based on N_2_ adsorption/desorption analysis.

	Cathodes		
Characteristic	AC	CB	G
BET surface area (m^2^/g)	355.3996	177.8102	2.9440
t-Plot micropore area (m^2^/g)	146.2380	2.7059	0.5463
t-Plot micropore volume (cm^3^/g)	0.060737	0.000224	0.000041
BJH desorption cumulative volume (cm^3^/g)	0.278715	0.879021	0.037034
BJH desorption average pore diameter (nm)	5.6651	17.1297	31.2793

## Data Availability

Data will be made available on request.
